# Developing the infrastructure for patient review in academic journals

**DOI:** 10.1186/s40900-018-0114-2

**Published:** 2018-09-10

**Authors:** Sophie Staniszewska, Richard Stephens, Ella Flemyng

**Affiliations:** 1 Warwick Research in Nursing, University of Warwick Medical School, Coventry, UK; 2 Involved and engaged patient and carer, Stevenage, UK; 3 BMC part of Springer Nature, London, UK

**Keywords:** Patient involvement, Patient engagement, Peer review

## Abstract

**Plain English summary:**

Peer review is a well-established part of academic publishing. Its function is to assess the quality of a manuscript before publication in a journal. *Research involvement and Engagement* is the world’s first co-produced journal dedicated to developing the evidence base of patient and public involvement and engagement in health and social care research. Alongside traditional academic peer review we also involve other key stakeholders, including patients, carers, the public, policy makers, funders and practitioners. Following a recent survey looking at the motivations and feedback from patient reviewers in academic journals*,* we consider the key findings, reflect on what we already do and based on the feedback from the survey, we outline plans for future development. These plans include improving training and guidance for reviewers, changes to systems and workflows, acknowledging and engaging reviewers, and building a sense of community.

**Abstract:**

Peer review is a well-established part of academic publishing. Its function is to assess the quality of a manuscript before publication in a journal. *Research involvement and Engagement* is the world’s first co-produced journal dedicated to developing the evidence base of patient and public involvement and engagement in health and social care research. Alongside traditional academic peer review we also involve other key stakeholders, including patients, carers, the public, policy makers, funders and practitioners. Following a recent survey looking at the motivations and feedback from patient reviewers in academic journals*,* we consider the key findings, reflect on what we already do and based on the feedback from the survey, we outline plans for future development. These plans include including improving training and guidance for reviewers, changes to systems and workflows, acknowledging and engaging reviewers, and building a sense of community.

## Editorial

Peer review is a traditional and well-established part of academic publishing. Its function is to assess the quality of a manuscript before it is published. Submitted manuscripts are assessed by reviewers for originality, validity and significance, to help editors determine whether a manuscript should be published in their journal [1]. Peer review has existed for many years and while it can be flawed, it is the best available approach that we have, with most wanting to improve it rather than replace it [2, 3].

As the world’s first co-produced journal dedicated to developing the evidence base of patient and public involvement and engagement in health and social care research, *Research Involvement and Engagement (RIE)* has extended the concept of peer review beyond the confines of traditional academia, to draw on a broader range of people, including patients, carers, the public, policy makers, funders and practitioners. We believe this enhances the diversity of review and offers authors a broader evaluation of their work, through a wider assessment of relevance, acceptability, appropriateness and robustness, arguably a form of ‘community validity’ [4]. The academic and the patient peer reviewers provide different, but complementary evaluations, and as Editors we treat their reviews as equally important, recognising the intrinsic contribution each makes to the assessment of a manuscript.

If the publishing community is to embrace patient review as standard, then we need to understand how patients experience a publisher’s systems and processes, so we can ensure a high quality experience for patients, as well as for academics. Together with colleagues at the British Medical Association and *The British Medical Journal* (*BMJ*), who also include patients in peer review, we surveyed patient peer reviewers who had completed reviews for both *RIE* and the *BMJ* [5]. In this Editorial we consider key findings from that survey, we reflect on what we already do and based on the feedback from the survey, we outline plans for future development.

## Patient reviewer survey results and feedback

Across both journals, the key motivation of patients to review was the opportunity to add the patient or carer perspective to an article (*n* = 157, 90% felt this was either 'extremely' or 'very' important). Of the respondents, 81% (*n* = 157) would recommend being a patient reviewer to other patients and carers, and 92% (*n* = 157) thought more journals should adopt patient review. Survey questions on patient reviewers’ perceptions of open peer review process highlighted that 81% (*n* = 224) had no concerns with open peer review, 5% had concerns and 7% were unsure [5].

From the survey, we identified four important areas for us to develop. They are improving training and guidance, changes to systems and workflows, acknowledging and engaging reviewers, and building a sense of community.

## Improving training and guidance

Confidence in reviewing emerged as a challenge in how patients experienced peer review, 27% (*n* = 157) reported feeling either not at all or only slightly confident when doing their first review. This proportion fell to 8% (*n* = 141) for those who had done more than one review. Overall, the survey highlighted that only 61% (*n* = 157) of patient reviewers found the reviewer instructions ‘extremely’ or ‘very helpful’ (for *RIE* reviewers this was 51%). The feedback highlighted many ways in which these could be improved, including more guidance on how to conduct a review, diagrams or videos of the submission process that explain what to expect at different stages, and sample reviews for reference [5].

Based on this feedback, improving our training and guidance is an important area for us to develop, as we want our reviewers to be as confident as possible when conducting a review for the journal. In response to the survey we have redeveloped the *RIE* reviewer guidelines [1] ensuring that we captured the areas that patients wanted more clarity on; this includes what peer review is and how it works in the journal, links to training resources for reviewers, highlighting that existing example reviews are publically available through our open peer review policy, information on what to consider when you receive a reviewer invite, and points to consider when writing a review. These were developed with feedback from our Editorial Board, including some of our patient, carer and public representatives. Within *RIE*, academics and patients are treated equally and these guidelines have been developed, and are used, by both our academic and patient reviewers, which is fundamental for a co-produced journal. We will also continue to update the guidance as we receive feedback.

**Fig. 1:**
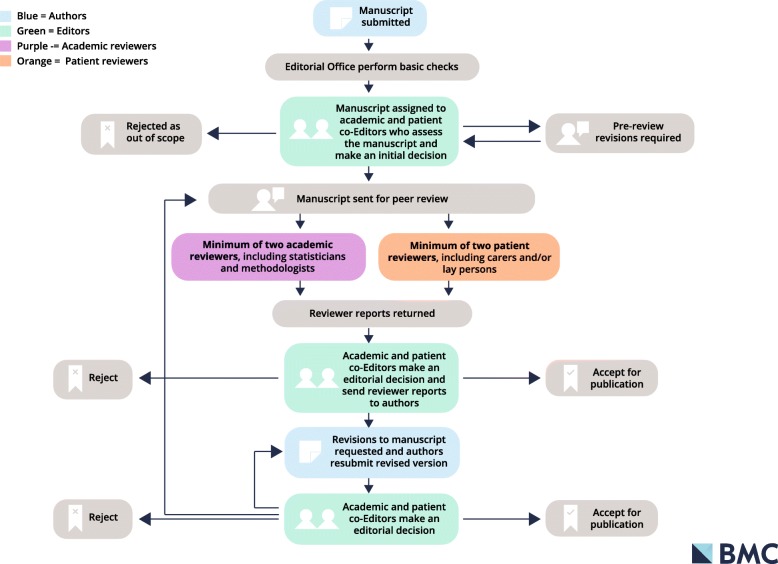
Example of the manuscript workflow following submission by an author *Research Involvement and Engagement*

Our reviewer guidelines now have a prominent place on the journal homepage so people can access it more easily. To help patient reviewers understand the wider publishing process we have also developed a manuscript workflow image that summarises what happens when an author submits to the journal, which is available on our reviewer guidelines page (see Fig. 1).

Within the survey patients highlighted that they would like more support, encouragement, training and forums that enable them to ask questions. Some patients highlighted peer-to-peer mentoring, which some journals have recently begun implementing, such as *Trials* [6].

In the longer term, areas we feel are important for future development include assessing how peer-to-peer mentoring might work within a journal like *RIE*, adding a frequently asked questions section to the reviewer guidelines, and to investigate tools for collaborative peer review.

## Changes to systems and workflows

There was a range of feedback on how the system and workflows could be improved. Some are unfortunately not possible due to limitations with editorial software, some are already possible and has highlighted that we need to improve our guidance to ensure this is clear, and some we will investigate further.

For example, some patients wanted more time to review so we will ensure it is clear that reviewers can ask for an extension in the deadline to submit a review; we prefer people do this over declining to review. Patient reviewers also wanted better matching of the content of manuscripts to reviewers’ experience. Last year we redeveloped the classifications used within our editorial system to support better matching of reviewers to manuscripts. We encourage all reviewers to ensure their details within the editorial system are up-to-date and encourage them to also use personal keywords to help us find the right people for particular topic areas.

There were also important messages for us as Editors. Patients wanted feedback on the usefulness and benefits of their reviews, and how manuscripts have changed as a result to help them understand the value of the review. We are exploring different options for this and would also like to encourage authors to provide a few key points about how the patient review helped them develop their manuscript in their point-by-point response to reviewers, which authors sometimes provide when submitting a revised manuscript.

Patients also requested better sharing of information around decisions made on manuscripts they reviewed; all reviewers in *RIE*, both patient and academic, should receive a notification when a decision has been made on an article they reviewed. Feeling valued was important, and being asked to review again was central to this. From *RIE’s* perspective, this partly depends on the number of manuscripts submitted and what areas they fall in but is something we will monitor. The final key element was treating patient reviewers as equal partners in the peer review process. For *RIE* this is the essence of our mission and already embedded in the way we work, valuing the important and unique contribution patients bring to the review of research.

## Acknowledging and engaging reviewers

Improving the way we acknowledge and engage with our reviewers is essential for any journal as we would not survive without the generous time and insightful comments of our reviewers. For these reasons, *RIE* publicly acknowledges its peer reviewers on an annual basis [7].

Over recent years we have ensured that on an annual basis, following the posting of the reviewer acknowledgment list, that we send personal thank you emails to all reviewers from the previous year. We highlight in this email that we can provide reviewer certificates or confirmation of review on branded letterheaded paper, if reviewers require one for their own personal or professional records. We also invite some of our reviewers to become Editorial Board Members for a period, to help shape the journal going forward.

Within the survey, patients called for access to articles published in subscription journals. We understand that this would be of great benefit but as an open access journal, all articles published in *RIE* are freely available for anyone to read. Instead, if our patient reviewers are interested in submitting manuscripts to the journal and have submitted a certain number of reviewer reports in the last year, they can request a waiver for the Article Processing Charge associated with publishing in the journal. It was promising to hear from the survey that at least one patient reviewer from the survey had used this waiver to publish an article from their patient group [5].

There were a range of other suggestions for acknowledging reviewers, which are fully detailed in the survey [5]. We are considering many of these, for both patient and academic reviewers, and will be sure to share more information on developments when they are available.

## Building a sense of community

The final area identified focused on building a sense of community, which is the most challenging area, given that reviewing by its nature is a solitary activity, and our reviewers are spread across the globe. We plan to explore opportunities for more tangible ways of creating a sense of community by potential collaborations with key organisations leading on involvement and engagement internationally. One possibility is the newly formed International Patient and Public Involvement Network [8].

## In conclusion…

Patient peer reviewer is a cornerstone of *RIE*, capturing the essence of collaboration and the co-production of knowledge (Table 1). As one of our respondents said, we want to “raise the profile of patient review by illustrating the value added and changes made” [5]. Already other academic journals have approached us to explore how they could include patients in peer review.

**Table 1:** *Research Involvement and Engagement’s* position on patient peer review

• Academic and the patient peer review provide different, but complementary evaluations, and we recognise the intrinsic contribution each makes to the assessment of a manuscript. • We believe incorporating both academic and patient review enhances the diversity of review and offers authors a broader evaluation of their work, through a wider assessment of relevance, acceptability, appropriateness and robustness, arguably a form of ‘community validity’. • We are dedicated to treating patient reviewers as equal partners in the peer review process recognising their unique contribution. For *RIE* this is the essence of our mission and already embedded in the way we work.	

As Editors of *RIE* we place equal value on academic and patient peer review, but there is still much to understand about the way in which patient peer review strengthens research. Our joint survey with the *BMJ* forms a key stepping stone in our journey, helping us understand how we support and develop patient peer review. Going forward, we want to strengthen our understanding of the contribution and impact of patient peer review. Such evidence will help drive the paradigm change required in academia, enabling research to orientate itself towards the co-production of high quality, relevant and impactful knowledge that truly creates patient and public benefit.

## Abbreviations

*BMJ*: *The British Medical Journal*
*RIE*: *Research Involvement and Engagement*
